# Role of oxidative stress in epileptogenesis and potential implications for therapy

**DOI:** 10.1007/s43440-020-00143-w

**Published:** 2020-08-31

**Authors:** Kinga K. Borowicz-Reutt, Stanisław J. Czuczwar

**Affiliations:** 1 Independent Unit of Experimental Pathophysiology, Medical University of Lublin, Lublin, Poland; 2 Department of Pathophysiology, Medical University of Lublin, 20-090 Lublin, Poland

**Keywords:** Free radicals, Oxygen species, Nitrogen species, Oxidative stress, Epileptogenesis

## Abstract

In a state of balance between oxidants and antioxidants, free radicals play an advantageous role of “redox messengers”. In a state of oxidative stress, they trigger a cascade of events leading to epileptogenesis. During this latent, free of seizures period, a cascade of neurological changes takes place and finally leads to spontaneous recurrent seizures. The main processes involved in seizure generation are: neuroinflammation, neurodegeneration with anomalous neuroregeneration and lowering seizure threshold. Time of epileptogenesis offers a unique therapeutic window to prevent or at least attenuate seizure development. Animal data indicate that some antioxidants (for instance, resveratrol) may bear an anti-epileptogenic potential.

## Introduction

In both experimental and clinical conditions, antiseizure drugs (ASDs) were generally found disappointing in the aspect of neuroprotection and prevention of epileptogenesis [1–5]. Therefore, searching for not only antiseizure but also anti-epileptogenic strategies may become a new promising possibility to overcome problems associated with the treatment of drug-resistant epilepsy.

Production of reactive oxygen (ROS) and nitrogen species (RNS) is unavoidable even under physiological conditions. By definition, free radicals are atoms or their groups containing at least one unpaired electron on the outer shell. Molecular oxygen reduced by one electron makes superoxide (O2^*−•*^). One electron more leads to hydrogen peroxide (H_2_O_2_) generation, which is not a free radical, but when reduced to hydroxyl peroxide (HO*•*), it becomes the strongest oxidant currently known [6]. Produced superoxide can react with nitric oxide to form a very reactive peroxynitrite (ONOO* −*), another source of hydroxyl radical [6–8].

The mitochondrial respiratory chain provides production of around 90% of all free radicals. Other enzymes involved in the oxidative stress are: microsomal cytochrome P450 enzymes, flavoprotein oxidases, peroxysomal fatty acid oxidases [9]. Particularly important seem to be NADPH oxidase (Nox2), increasing superoxide production, and cyclooxygenase-2 (COX-2), stimulating astrocytes to synthesize proinflammatory cytokines [3]. Presently, Nox2 is considered as the main source of ROS at initial stages of epileptogenesis and neurodegeneration [10, 11].

Oxidative stress, understood as an imbalance between pro- and antioxidants, may prove highly harmful to cells, causing their damage and death. The first evidence that oxygen species result in tissue toxicity dates from 1954 [12]. In excess, free radicals can oxidize or nitrate DNA, proteins and fatty acids, eventually changing their structure and function. Cross-linking of base pairs in the DNA chain leads to gene mutations [3, 13–17].

In the central nervous system, oxidative/nitrative stress and neuroinflammation underlie the pathogenesis of neurodegenerative diseases, stroke- and age-related dementia, carcinogenesis and epilepsy [3, 6, 16, 17]. Brain is particularly sensitive to the action of free radicals. Furthermore, activity of enzymatic antioxidants, like catalase or glutathione peroxidase, is around ten times lower in the brain [3, 13]. Consequently, produced free radicals may oxidize and impair the function of neurotransmitters, like serotonin, dopamine or norepinephrine. Neuronal membranes, rich in polyunsaturated fatty acids, become a target for lipid peroxidation, resulting in decreased membrane fluidity and altered membrane transport [3, 5, 13, 18].

## Antioxidative mechanisms

Natural protection against free radicals is provided by enzymatic and non-enzymatic mechanisms. Enzymatic antioxidants are considered to be more specific and effective. Superoxide dismutase (SOD) catalyzes conversion of superoxide to hydrogen peroxide and molecular oxygen (2O_2_^•^– + 2H^+^  → H_2_O_2_ + O_2_). The copper–zinc SOD (CuZnSOD) acts in the cytoplasm and peroxisomes, while the manganese form (MnSOD) in mitochondria. Extracellularly acting ECSOD also contains copper and zinc [7].

Catalase breaks down hydrogen peroxide to molecular oxygen and water (2H_2_O_2_ → O_2_ + 2H_2_O). Hence, catalase does not eliminate free radicals, but protects conversion of hydrogen peroxide to hydrogen radical [7].

Glutathione peroxidase, a selenoflavoprotein, catalyzes reactions between hydroperoxides (including hydrogen peroxide) and reduced glutathione (GSH) to oxidized glutathione (GSSG) and water (ROOH + 2GSH → ROH + GSSG + H_2_O). Glutathione peroxidase exists in the cytoplasm, cell membranes and extracellularly. Another selenoflavoprotein (thioredoxin reductase) and glutaredoxin reduce GSSG, thus increasing GSH reserves. Additionally, thioredoxin reductase regenerates the oxidized form of ascorbic acid [3, 5, 8, 13, 17].

Non-enzymatic antioxidants, like ascorbic acid (vitamin C), α-tocopherol (vitamin E), β-carotene (a precursor of vitamin A), flavonoids and some trace elements, are exogenous substances supplied with diet. Ascorbic acid is a hydrophilic substance located in the cytoplasm that removes superoxide and hydroxyl radicals, as well as reduces the oxidized α-tocopherol. At higher levels, ascorbic acid may behave as a pro-oxidant promoting hydroxyl generation. α-Tocopherol, a lipophilic substance located in cell membranes, increases catalase activity and (together with GPH) decreases lipid peroxidation. This antioxidant also scavenges peroxyl radicals, which are converted to a free radical (tocopheryl) in this process. Then, tocopheryl is re-transformed to α-tocopherol due to the action of ascorbic acid. Again, at higher concentrations, tocopheryl radicals may contribute to cell damage. β-Carotene and flavonoids eliminate superoxide and lipid-derived radicals. Flavonoids seem to be more potent than remaining exogenous antioxidants. Finally, trace elements (copper, zinc, selenium) serve as co-factors of enzymatic antioxidants. In turn, GSH, an endogenous tripeptide, serves as a co-factor of glutathione reductase. This enzyme maintains the proper redox status of sulfhydryl proteins, involved in DNA expression and repair processes. Coenzyme Q10 is an endogenous lipid ubiquinone found in the inner mitochondrial membrane respiratory chain and transferring electrons from NADH dehydrogenase to the cytochrome bc_1_. It participates in reduction of superoxide to water [5–7, 13].

## Oxidative stress and mitochondrial dysfunction

Mitochondrial dysfunction induced by oxidative stress is markedly involved in neurodegeneration and epileptogenesis. In rodents, recurrent seizures induce excessive mitochondrial production of free radicals, above all superoxide, which are then converted to hydroxyl species. The latter in the presence of Cu^2+^ and Fe^2+^ oxidize lipids, proteins and DNA, resulting in impaired neuronal metabolism and gene expression, increased membrane permeability and excitability leading finally to decreased seizure threshold [3, 9]. Dysfunctional mitochondria enhance ROS production and processes leading to both apoptotic and necrotic cell death. Experimental and clinical data confirm increased oxygen and glucose uptake in the brain following the first seizure [3, 13, 18]. Lipid peroxidation may also be induced by phospholipase A2 that releases arachidonic acid from cell membranes. Arachidonic acid metabolites trigger lipid peroxidation resulting in production of conjugated aldehydes. They, in turn, activate the caspase cascade prompting the apoptotic cell death [5]. In contrast, NO at low levels was reported to inhibit apoptosis through nitrosation of caspases [15].

The main mitochondrial antioxidant is MnSOD. Sirtuin 3 is a class III histone deacetylase, increasing activity of the mitochondrial electron chain and subsequent ATP production through β-oxidation process. Additionally, it enhances MnSOD action through deacetylation of the Lys 122 residue. On the experimental level, sirtuin 3 deficiency generated oxidative stress and severe seizures in MnSOD deficient mice. Hippocampal neurodegeneration in vitro was also observed [13, 19].

Mitochondria also maintain a stable level of intracellular calcium; therefore, mitochondrial dysfunction leads to calcium overload. The combined effect of ROS and calcium overload increases synaptic transmission, neuronal excitability and excitotoxicity. It leads also to opening of mitochondrial permeability transition pores and translocation of proapoptotic molecules to cytosol, which initiates apoptotic cell death. After the first seizure, neurodegeneration is predominantly observed in CA1/CA3 hippocampal regions. Considerable neuronal death in the CA3 subfield was observed simultaneously with decreased GSH/GSSG ratio and increased mitochondrial peroxidation within 2–7 days following kainate injection. Increased ROS generation in CA1, CA3, and the dentate gyrus combined with neural death in these areas was noticed also in rats after pilocarpine-induced seizures [9, 13].

Hippocampal cell death is implicated not only in epileptogenesis, but also in cognitive dysfunction observed in normal aging, neurodegenerative diseases and epilepsy [19]. It should be remembered, however, that both oxidative stress and neuroinflammation may be not only reasons, but also consequences of seizures. That is why seizures can induce further seizures [18, 20, 21].

## Oxidative stress and epileptogenesis

**Fig. 1:**
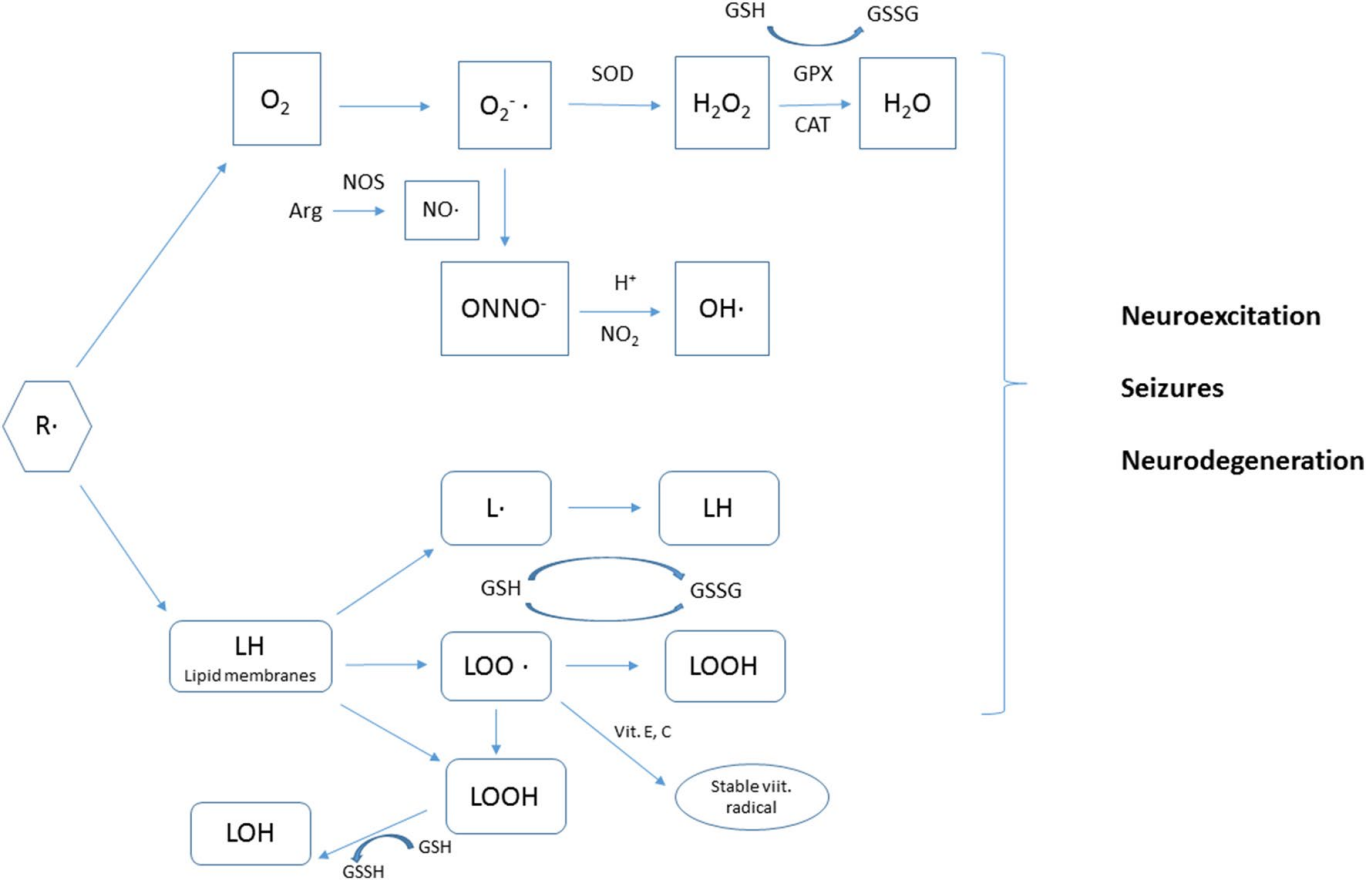
The outline of oxidative stress processes. *R*· free radical, *O*_*2*_^−^· superoxide, *NO*· nitric oxide radical, *ONNO*· peroxynitrite, *OH*· hydroxyl peroxide, *L*· lipid radical, *LOO*· lipid peroxy radical, *LOOH* lipid hydroperoxide, *LOH* stable lipid alcohol, *NOS* nitric oxide synthase, *SOD* superoxide dismutase, *GSH* glutathione, *GSSG* oxidized glutathione, *GPX* glutathione oxidise, *CAT* catalase

**Fig. 2:**
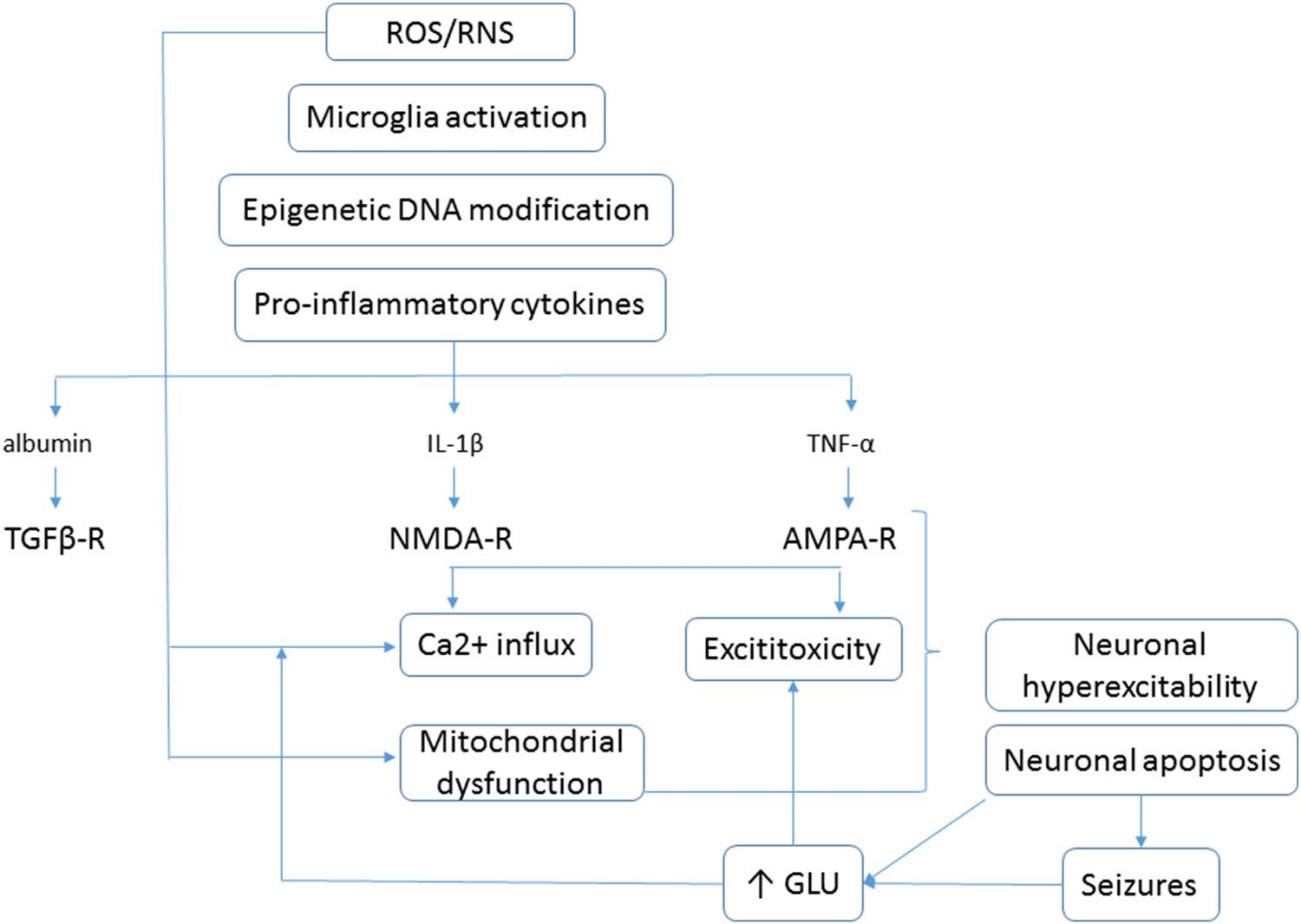
Oxidative stress and neuroinflammation in pathogenesis of seizures. *ROS* reactive oxygen species, *RNS* reactive nitrite species, *TGFβ-R* transforming growth factor β receptor, *NMDA-R* *N*-methyl-d-aspartate receptor, *AMPA-R* α-amino-3-hydroxy-5-methyl-4-isoxazolepropionic acid receptor, *IL-1β* interleukin 1β, *TNF-α* tumor necrosis factor α, *GLU* glutamate

ROS/RNS, neurodegeneration, neurogenesis and lowering the seizure threshold are recognized as the most important hallmarks of epileptogenesis [9, 20]. Data about this process come mainly from experimental models of partial complex seizures and autopsy of patients with temporal lobe epilepsy. Presently, kainate- or pilocarpine-induced status epilepticus in rats is recognized as the most reliable models of epileptogenesis (Figs. 1, 2).

Epileptogenesis is defined as free from seizures latent period between the initial brain insult and the first unprovoked seizure. At that time, a cascade of neurological changes takes place and finally leads to spontaneous recurrent seizures. This latent period, lasting up to even several years, offers a therapeutic window to prevent or at least attenuate seizure development. The most frequent triggering factors in patients with temporal lobe epilepsy are traumatic brain injury, stroke, intracerebral infections, and intoxications [3].

In details, epileptogenesis is a time of very intensive neuronal alterations, including excessive production of ROS/RNS and pro-inflammatory mediators, neurodegeneration, excessive anomalous neurogenesis, reorganization of survived neuronal circuits, maladaptive dendritic plasticity, axonal sprouting within excitatory pathways accompanied by loss of inhibitory interneurons, reactive gliosis, damage to the blood–brain barrier, re-arrangement of the extracellular matrix and neuronal cytoarchitecture. As a result, increased brain excitability leads finally to neuronal hypersynchronization, increased epileptiform spiking and spontaneous recurrent seizures [22, 23].

A close relationship between two hallmarks of epileptogenesis (neuroinflammation and redox status imbalance) was evidenced in the mouse model of temporal lobe epilepsy induced by Theiler’s murine encephalomyelitis virus. Neuroinflammation is manifested in intense astrogliosis and microgliosis. Astrocytes, activated by nuclear factor erythroid regulated factor 2 (Nrf2, a cytoplasmic transcription factor), produce antioxidants, such as superoxide dismutase, catalase, glutathione peroxidase, and GSH [21].

The seizure insult entails also dysfunction of glutamate transporters (GLT-1, GLAST) in astrocytes, which is considered as a crucial pathogenic factor of human epilepsy. Accumulated glutamate is then released outside through calcium-dependent mechanism. Glutamine synthase transforms glutamate to glutamine. After an initial seizure, glutamine synthase activity transiently increases in nonreactive astrocytes, but decreases again in reactive astrocytes during the latent phase of epileptogenesis. Excessive amounts of non-metabolized glutamate get into the extracellular space, increasing neuronal excitability and evoking spontaneous recurrent seizures [3].

Moreover, reactive astrocytes are an important source of pro-inflammatory mediators, such as IL-1β, IL-6, IL-10, TNFα and interferons. Pleiotropic actions of ROS/RNS and pro-inflammatory cytokines contribute significantly to the development of epilepsy [3]. Interleukin-1β (IL-1β) and tumor necrosis factor α (TNFα) can alter excitability through modulating receptor expression and function. IL-1β stimulates NMDA, while TNFα on AMPA receptors. Increased Ca^2+^ influx through glutamate receptors contributes to neuronal hyperexcitability and degeneration [23]. IL-6, fibrinogen and high-sensitivity C-reactive protein were found in serum, cerebrospinal fluid and brain of patients with epilepsy. Overexpression of IL-1β, IL-6, COX-2, TNFα and inflammatory transcription factor nuclear factor kappa-light-chain-enhancer of activated B cells (NFκΒ) was detected in the hippocampal and piriform cortex in the pilocarpine model of status epilepticus in rats. NF-κB, quintessential for neuronal plasticity and memory functions, is activated by pro-inflammatory cytokines (IL-1β, IFN-γ, TNF-α) and increased excitatory synaptic transmission [24]. Interestingly, changes in cytokine expression correlated well with seizure activity. Levels of pro-inflammatory mediators were the highest just after the initial insult, returned to normal values during the latent period, and raised again in the phase of chronic seizures. This pattern coincides with changes in ROS concentrations, the degree of mitochondrial damage and apoptotic cell death. In turn, apoptosis aggravates inflammatory responses [5, 21].

One of neuroinflammatory mediators is Fyn, a member of Src family of tyrosine kinase (SFK) and a protein regulating cell growth. Experimental data showed that suppressing Fyn pathway decreases inflammation and hippocampal RNS production. Saracatinib, an SFK inhibitor, was reported to prevent epileptogenesis in 50% of rats after kainic acid-induced status epilepticus. In remaining animals, it reduced spontaneous recurrent seizures and epileptiform spiking [20]. Also thyroid hormones play an essential role in epileptogenesis and neuroregeneration. Molecular studies evidenced that deficiency of thyroid hormones causes mitochondrial damage, generates oxidative stress and impairs the function of GABAergic neurons [25].

Astrocytes are an integral component of the blood–brain barrier. In response to brain injury, astrocytes enhance production of the endopeptidase matrix metalloproteinase (MMP-9) that further compromises the blood–brain barrier and makes it highly permeable to fluid and blood proteins. In turn, albumins activate astrocytes, through the p38 MAPK pathway, to produce more inflammatory mediators [26]. On the other hand, astrocytes, being close to synaptic terminals and blood vessels, tend to support neurons by up-taking the extra synaptic glutamate and potassium and by providing them with glucose [20].

Nonreactive microglia, the resident macrophages in the brain, are mainly responsible for cell debris engulfment. After the initial seizure insult, microglia are involved in neuroinflammation, neuroprotection, neurogenesis and neurodegeneration processes. These secrete neurotrophic, anti-inflammatory and cytotoxic factors, as well as activate Nox2 complex and increase ROS production [21, 22]. Activated microglial cells move to the place of injury and release tissue plasminogen activator (tPA), a marker of chronic epilepsy involved in the process of mossy fiber sprouting [3]. In epileptogenesis, the microglia become activated around 30 min following an initial insult and migrate to the synaptic terminals. There, they are engaged in dendritic pruning [27, 28] and support neuronal survival by producing trophic agents [29].

The first type of epilepsy proved to be related with oxidative stress was myoclonic epilepsy with ragged red fibers. Its causative factor is a mutation in mitochondrial DNA that leads to increased ROS followed by the respiratory chain damage and decreased ATP generation [30].

In preclinical studies, increased concentration of ROS/RNS and attenuated GSH action were observed in the pilocarpine and kainate models of temporal lobe epilepsy [31]. In rat brains, kainate-induced status epilepticus was followed by the tricyclic acid cycle disorders manifested by diminished accessibility of reducing factors (NADH, NAD cytochrome c reductase, FADH2) and impaired ATP synthesis. Additionally, kainic acid increases intracellular Ca^2+^ influx activating variety of enzymes, including nitric oxide synthase (NOS). Induced nitrative stress initiated an extensive lipid peroxidation with subsequent damage of membranes and organelles. Except hippocampal apoptosis, altered gene expression and protein synthesis were observed in survived neurons [3, 32]. In the same model, nNOS/iNOS upregulation and a biphasic changes in nitric oxide and 3-nitrotyrosine (3NT) hippocampal levels were observed. NO and 3NT concentrations increased rapidly after induced SE, then lowered to control values 2 days post SE and increased again, and remained elevated for 6 weeks coinciding with the epileptogenic period [20].

Pilocarpine, another chemoconvulsant, initiates an intensive production of superoxide and hydrogen peroxide in the latent period of epileptogenesis and the phase of chronic epilepsy [5, 7, 18, 21].

In posttraumatic epilepsy, where a triggering point is blood extravasation, liberation of Fe^3+^ from erythrocytes induces superoxide, hydroxyl and hydrogen peroxidase production. ROS-induced lipid peroxidation and the resulting membrane disintegration are followed by increased secretion of excitatory amino acids. Moreover, hydroxyl radicals induce production of methylguanidine, an endogenous convulsant derived from creatinine [33, 34]. RNS-induced posttranslational modifications may also contribute to the development of the posttraumatic epilepsy [33].

## ROS and lowering the seizure threshold

Brain concentrations of hydrogen peroxide change over time; they increase after the initial insult, then, they are reduced by the action of antioxidants in the latent period, and again enhanced during spontaneous recurrent seizures because of depletion of antioxidants. Simultaneously, excessive ROS generation results in mitochondrial DNA damage and decreased activity of tricyclic acid cycle enzymes. ROS-induced change in glutamate receptors structure, decreased energy-dependent action of glutamate transporters and loss of GABA-ergic neurons in the hippocampus, and dentate gyrus finally increases neuronal excitability and seizure susceptibility [3, 13].

## Co-existence of oxidative stress and inflammation in epileptogenic events

Oxidative stress and neuroinflammation often co-exist in brains of patients with different types of epilepsy and some epileptogenic cortical malformations. NF-κB was reported to be a factor switching an inflammatory state after oxidative stress. In fact, low level of oxidative stress stimulates NF-κB, creating a kind of equilibrium between this factor and two associated regulatory proteins: SHIP-1 and SOCS-1. Such a state does not induce expression of inflammatory genes. However, high level of oxidative stress additionally leads to increased IL-1β and toll-like receptor 4 (TLR4) expression which reinforces NF-κB signaling further. In this case, SOCS-1 cannot balance, creating a vicious circle of a self-sustaining inflammatory signal. Moreover, TLR4 was probed to be crucial in ictogenesis and seizure development processes. These findings encourage conclusion that intensity of oxidative stress can predict the possibility of epileptogenesis and, second, antioxidant therapy (e.g. inhibitors of ROS-inducing enzymes) and/or inhibition of cyclooxygenase may prevent some epileptogenic processes in the brain [35, 36].

## Antiseizure drugs and epileptogenesis

Antiseizure drugs (ASDs) are classified by the “generation” in which they were introduced. The era of the first-generation ASDs started from phenobarbital, which is still widely used in the prophylaxis of febrile seizures in children and in a veterinary practice. Representatives of benzodiazepines (clonazepam, diazepam, and lorazepam) are applied most often in the treatment of status epilepticus. Another first-generation ASD, ethosuximide, is indicated for the treatment of absence epilepsy. Phenytoin is, in turn, indicated for the prevention of seizures accompanying neurosurgery and the control of generalized tonic–clonic status epilepticus. The last of the first-generation ASDs, valproate, is prescribed for all seizure types including absence, myoclonus and tonic–clonic seizures. Second-generation ASDs include 11 approved medications: vigabatrin, oxcarbazepine, lamotrigine, gabapentin, topiramate, tiagabine, levetiracetam, pregabalin, zonisamide, stiripentol and rufinamide. Whereas, the most known examples of third-generation ASDs are brivaracetam, eslicarbazepine, lacosamide, losigamone and retigabine. When compared to first-generation ASDs, the second- and third-generation did not prove to be more effective. However, many of them are better tolerated and less often interact with other drugs. Most of them are approved for adjunctive therapy of epilepsy [37].

First-generation ASDs occurred rather disappointing in the aspect of neuroprotection and prevention of epileptogenesis, valproate showing some antiepileptogenic potential [1–5]. Moreover, this class of ASDs often enhances epilepsy aftermaths, impairing cognition and decreasing mood [32, 33, 38]. Among second-generation ASDs, gabapentin showed an antiepileptogenic effect, while vigabatrin, tiagabine, lamotrigine and topiramate remained ineffective in this respect [4].

Such discouraging results may be related to weak influence of ASDs on the cell redox balance. Some antioxidant activity was found for gabapentin [39] and zonisamide, which exhibited significant antioxidant action, inhibiting lipid peroxidation, decreasing production of nitric oxide and scavenging hydroxyl radicals [33]. Antioxidant properties of zonisamide contribute to the neuroprotective characteristics of this drug [9]. However, reports about first-generation ASDs seem to be very contradictory. In kindled rats, phenobarbital, diazepam and valproate showed a moderate antioxidant action, phenytoin was even less effective, while carbamazepine remained entirely ineffective. In one study, phenytoin did not affect lipid peroxidation. In others, valproate, phenytoin, carbamazepine and levetiracetam enhanced this process. Additionally, also reactive metabolites of several classical ASDs (e.g. phenobarbital, phenytoin, carbamazepine, valproate) can induce ROS production and contribute to cell damage [18].

In some experimental and clinical studies, carbamazepine reduced catalase activity, whereas valproate and carbamazepine lowered superoxide dismutase expression [3, 11]. In contrast, 6- and 12-month valproate monotherapy increased activity of antioxidant enzymes (glutathione reductase, glutathione peroxidase, superoxide dismutase, and catalase) in 32 pediatric patients. Simultaneously, reduced levels of oxidative markers, such as malondialdehyde, hydrogen peroxide, 8-hydroxy-2-deoxyguanosine and 3-nitrotyrosine, were observed [1].

## Antioxidants with an antiepileptogenic potential?

The combined treatment with 4-(2-aminiethyl)-benzensulfonyl fluoride (AEBSF, a Nox2 inhibitor) and a cyanoenone triterpenoid (RTA 408) activated Nrf2 and decreased ROS production, mitochondrial dysfunction and neuronal death in in vitro model of seizure-like activity. Furthermore, it prevented spontaneous recurrent seizures development in 70% of rats after kainate-induced status epilepticus [10]. In this model, also alpha-tocopherol showed some antiepileptogenic properties [40]. Specifically, pretreatment with this agent prevented changes in the number of population spikes generated by pyramidal cells and reduced neuroinflammation. Also, a strong neuroprotection was evident as well as normalization of the blood/brain barrier [40].

Pre-treatment with ascorbic acid showed neuroprotective and anticonvulsant effects in the pilocarpine-, pentetrazole- and penicillin-induced seizures in rodents, remaining ineffective in the kainate model. The antiepileptogenic/anticonvulsant action was reflected by increased latency to spontaneous recurrent seizures, decreased mean seizure score and reduced mortality. All these observations have been attributed to reduced lipid peroxidation and increased CAT activity [9]. Effectiveness of α-tocopherol in neuroprotection and mitigating seizures in humans and animal models may be due to reduction of the oxidative stress, regulating gene expression and inhibiting neuroinflammation. The latter is indicated by decreased levels of pro-inflammatory cytokines (IL-1β, TNFα) and increased anti-inflammatory IL-6. In turn, IL-6 was found to protect against glutamate and NMDA-induced toxicity and enhance purinergic transmission through up-regulating gene expression for adenosine A_1_ receptors [40]. Furthermore, α-tocopherol was reported to reduce astrogliosis and microglial activation, restore activity of glutamine synthase in astrocytes and induce synaptogenesis. These mechanisms may contribute to anticonvulsant effects of α-tocopherol in animal models of posttraumatic epilepsy and generalized tonic–clonic convulsions [11, 33]. Additionally, α-tocopherol, applied to rats during the latent phase after KA-induced SE, normalized pyramidal cell electrical activity and latency to the onset of seizure-like activity in vitro, as well as decreased the rate of hippocampal neurodegeneration in kindled rats [11].

Supplementation of coenzyme Q10 prevented RNA oxidation, seizure development and neuronal loss in rats undergoing pilocarpine-induced status epilepticus [5–7, 13].

Melatonin, a potent antioxidant, scavenges hydroxyl radicals, increases the action of glutathione peroxidase, inhibits NOS activity, decreases ROS production and lipid peroxidation, reverses mitochondrial dysfunction and reduces hippocampal cell loss [33]. Anticonvulsant and neuroprotective actions of melatonin were evidenced in both experimental and clinical studies. Interestingly, pinealectomy has been reported to induce convulsions. In rat models of temporal lobe epilepsy, melatonin reduced cell death, mossy fiber sprouting, lipid peroxidation and microglial activation [40, 41]. Moreover, this hormone mitigated iron-induced seizures, increased the latency of penicillin- and pilocarpine-induced seizures. Melatonin was also reported to reduce deleterious effects of kainate-induced status epilepticus on nuclear and mtDNA [9]. In intractable epilepsy patients, melatonin reduced spiking activity and seizure frequency [32]. Antiseizure effects of melatonin have been attributed to the up-regulation of GABA_A_ receptors and inhibition of glutamate-mediated responses through decreasing nNOS activity [18].

Resveratrol, a polyphenol, reduces ROS generation through suppressing mitochondrial complex II activity and cytochrome c leakage. Moreover, it negatively modulates N-methyl-D-aspartate (NMDA) and kainate receptors, decreasing intracellular Ca^2+^ influx. Indirectly, resveratrol activates sirtuin 1, a class III histone deacetylase, which reduces inflammation, oxidative stress and apoptosis [15]. Anti-inflammatory action of this polyphenol is also mediated by the inhibition of mTOR pathway. Subsequent inactivation of NFκΒ decreases pro-inflammatory cytokine production. Resveratrol was widely examined in vitro and in numerous animal seizure models, like kainate- or pilocarpine-induced status epilepticus, pentetrazole kindling and posttraumatic epilepsy [33]. The main observed effect was suppression of the oxidative stress, neuroinflammation, neurodegeneration, astro- and microgliosis, mossy fiber sprouting, and spontaneous recurrent seizures. Importantly, resveratrol exhibited antiseizure and neuroprotective properties even when was given several days after chemoconvulsant application [38].

α-Lipoic acid, a cofactor for mitochondrial enzymes, was reported to increase superoxide dismutase, catalase and glutathione peroxidase activity. Moreover, it decreases nitrite generation and lipid peroxidation level. In rodents, α-lipoic acid showed anticonvulsant and neuroprotective potential in the pilocarpine-induced SE in rats [18] and ferric chloride-induced seizures, remaining without significant effects in the pentetrazole- and kainate models [9].

Curcumin, the main active component of turmeric, exhibits anti-inflammatory, antioxidant and neuroprotective properties in vitro, but not in the pilocarpine model of temporal lobe epilepsy [42]. This substance, applied to rats during the latent period after kainate-induced status epilepticus, did not prevent spontaneous recurrent seizures development, but ameliorated their severity. In the same model, curcumin ameliorated spatial memory and explorative behavior impaired by phenobarbital, carbamazepine and phenytoin [2, 11]. Curcumin is an effective scavenger of ROS/RNS, decreasing lipid peroxidation, astrocytic activation, oxidative DNA damage, mitochondrial dysfunction, and apoptotic cell death in the hippocampus [11]. Such effects can limit negative consequences of experimental seizures [2]. The most probable mechanism of action is inhibition of the mTOR and MAPK pathways [42].

Sitagliptin, an antidiabetic drug against diabetes type II showing also antioxidative properties, was tested against pentetrazole-induced acute epileptogenesis and the results are quite encouraging. The drug was started a week prior to pentetrazole and it efficiently inhibited the process of epileptogenesis along with potent neuroprotection and normalization of pentetrazole-induced biochemical disturbances in hippocampus: decreased levels of GABA and increased levels of glutamate, glial fibrillary acidic protein (GFAP) and brain-derived neurotrophic factor (BDNF) [43].

## Antioxidants in patients with epilepsy

The use of antioxidants as add-on therapy should protect from structural damages, epileptogenesis and cognitive deterioration in epileptic patients (Table 1).

**Table 1:** Effects of antioxidative agents on experimental seizures in rodents

Substance	Seizure model	Effects	References
AEBSF and	KA-induced SE	↓ SRS in 70% of animals	[10]
RTA 408	In vitro seizure-like activity	↓ ROS, ↓ mitochondrial dysfunction, ↓ neuronal death	[10]
α-Tocopherol	KA-induced SE	↓ Pyramidal cells-evoked spikes, ↓ neuroinflammation, ↓ neuronal death, BBB normalization	[40]
Ascorbic acid	PILO-, PTZ, PENI-induced seizures	↑ Latency to SRS, ↓ mean seizure score, ↓ mortality	[9]
Coenzyme Q10	PILO-induced SE	↓ Seizure development, ↓neuronal loss	[5–7, 13]
Melatonin	PILO-, KA-induced SE	↓ Cell death, ↓ mossy fiber sprouting	[40, 41]
	Fe-, PENI-, PILO-induced seizures	↑ Latency of seizures	[9]
Resveratrol	KA-, PILO-induced SE, PTZ kindling, posttraumatic seizures	↓ SRS	[33]
α-Lipoic acid	PILO-induced SE, Fe-induced seizures	Anticonvulsant	[9, 18]
Curcumin	KA-induced SE	↓ Severity of SRS	[2, 11]
Sitagliptin	PTZ-induced acute epileptogenesis	↓ Epileptogenesis, ↓ neuronal loss	[43]

*AEBSF* 4-(2-aminiethyl)-benzensulfonyl fluoride, *RTA 408* 4-(2-aminiethyl)-benzensulfonyl fluoride, *KA* kainite, *SE* status epilepticus, *PILO* pilocarpine, *PTZ* pentetrazole, *PENI* penicillin, *SRS* spontaneous recurrent seizures, *ROS* reactive oxygen species, *BBB* blood–brain barrier

α-Tocopherol (400 mg/day) was examined as an adjunctive drug in two randomized, double-blind, placebo controlled clinical trials carried out on patients with refractory epilepsy. In the first study, this antioxidant significantly decreased seizure frequency in 12/24 children, without influence on plasma concentrations of antiseizure drugs. Furthermore, in two independent trials, melatonin significantly decreased seizure frequency and improved quality of life in 5/10 and 5/6 pediatric patients, respectively. However, abovementioned observations were not confirmed in further studies [11].

## Conclusion

Oxidative stress plays a key role in epileptogenesis. The early effect of ROS/RNS action is reactive gliosis, lipid peroxidation and damage of mitochondrial DNA. All these processes result in neurodegeneration, neurogenesis, re-arrangement of neuronal circuits, hyperexcitability and decreased seizure threshold. Combination of antiseizure drugs with antioxidants may increase effectiveness of antiseizure treatment and/or limit epileptogenesis.
